# Both T and B cells are indispensable for the development of a PBMC transfer-induced humanized mouse model for SSc

**DOI:** 10.1186/s13075-022-02896-6

**Published:** 2022-08-25

**Authors:** Yaqing Shu, Xiaoyang Yue, Jacqueline Wax, Brigitte Kasper, Junping Yin, Xiaoqing Wang, Liang Zhang, Marjan Ahmadi, Harald Heidecke, Antje Müller, Peter Lamprecht, Xinhua Yu, Gabriela Riemekasten, Frank Petersen

**Affiliations:** 1 Priority Area Chronic Lung Diseases, Research Center Borstel, Member of the German Center for Lung Research (DZL), 23845 Borstel, Germany; 2 Department of Neurology, The Third Affiliated Hospital of Sun Yat-Sen University, Guangzhou, China; 3 Department of Histology and Embryology, School of Basic Medical Science, Guangxi Medical University, Nanning, Guangxi China; 4 CellTrend GmbH, Im Biotechnologiepark, 14943 Luckenwalde, Germany; 5 Department of Rheumatology, University of Lübeck, 23538 Lübeck, Germany

**Keywords:** Autoimmune diseases, Systemic sclerosis, Peripheral blood mononuclear cells, Autoantibodies, Systemic inflammation, B cells, T cells, Immunosuppressive drugs

## Abstract

**Background:**

Recently, a novel humanized mouse model for systemic sclerosis (SSc) was established by transferring peripheral blood mononuclear cells (PBMC) from patients with SSc to *Rag2*^*−/−*^*Il2rg*^*−/−*^ immunodeficient mice. Here, we aimed to investigate the role of T and B cells in this humanized mouse model.

**Methods:**

T and B cells were depleted in vitro from freshly isolated PBMC using anti-CD3 and anti-CD19 magnetic *microbeads*, respectively. Subsequently, PBMC and T or B cell-depleted PBMC were transferred into *Rag2*^*−/−*^*/Il2rg*^*−/−*^ mice via intraperitoneal injection. Twelve weeks after the transfer, mice were sacrificed and evaluated.

**Results:**

Mice transferred with whole PBMC from SSc patients developed systemic inflammation in the lungs, kidneys, and liver, and 6 out of 11 mice died or had to be sacrificed during the experiment. By contrast, such inflammation and death were not observed in mice transferred with corresponding T or B cell-depleted PBMC. In line with this finding, transfer with whole PBMC restored the splenic white pulp composing of human T, B, and plasma cells and led to the production of a considerable amount of human autoantibodies in recipient mice, while those immunological features were rarely observed in mice that received T or B cell-depleted PBMC. In contrast to our previous findings demonstrating a transfer of the protective effect of a B cell therapy into the mouse, treatment of SSc patients with chemical immunosuppressive drugs did not affect the pathogenicity of PBMC.

**Conclusions:**

This study demonstrates that both T and B cells are indispensable for the pathogenesis of the PBMC transfer-induced mouse model for SSc.

**Supplementary Information:**

The online version contains supplementary material available at 10.1186/s13075-022-02896-6.

## Background

Systemic sclerosis (SSc) is a severe connective tissue disease which is characterized by autoimmunity, inflammation, vasculopathy, and fibrosis [1]. Besides the involvement of the skin, many other inner organs are often affected in SSc. Histological studies indicate that such lymphocytic infiltrates are present at the early stages of the development of SSc, preceding the onset of fibrosis [2, 3]. Animal models, particularly mouse models, are powerful research tools which help us to understand the disease pathogenesis [4]. So far, more than twenty mouse models for SSc have been established, and they provide new insights into the understanding of the pathogenesis of the disease [5]. However, differences between mice and humans in many aspects, especially in the immune system, limit the translation from bench to bedside [6]. One strategy for overcoming this obstacle is to use humanized mouse models [7]. Recently, we have generated a humanized mouse model for SSc by transfer of peripheral blood mononuclear cells (PMBCs) from patients with SSc into immunodeficient mice [8]. After the transfer of PMBC from patients with SSc, mice generate several SSc-associated autoantibodies (abs) including anti-nuclear abs (ANA) as well as anti-angiotensin-II type 1 receptor (AT1R) and anti-endothelin-1 type A receptor (ETAR) abs. Moreover, mice developed lymphocytic infiltrations in the lungs, kidneys, liver, and muscles, mimicking the systemic inflammation in SSc. By contrast, abs and systemic inflammation are rarely observed in mice that received PBMC isolated from healthy subjects or patients with granulomatosis with polyangiitis [8].

Besides bridging the gap between mice and humans, PBMC transfer-induced humanized mouse model is featured by at least two advantages. On the one hand, PBMC can be purposely treated in vitro before the transfer, which allows to determine the role of candidate molecules or cells. On the other hand, the heterogeneity in the source of PBMC from SSc patients also provides an opportunity to investigate the effect of patient-associated factors on the disease manifestations. For example, our previous study showed that mice transferred with PBMC from SSc patients who were treated with rituximab, a B cell-depleting monoclonal antibody, neither produced abs nor developed systemic inflammation [8], suggesting an essential role of B cells in the disease pathogenesis. In this study, we aimed to investigate the role of human T and B cells in the humanized mouse model. For this purpose, the effect of B or T cell depletion from PBMC of SSc patients prior to their transfer into immunodeficient mice was analyzed in terms of disease development, mortality, and architecture of the lymphatic organs in the recipient animals. Based on the observed pivotal role of T and B cells, we have performed a retrospective analysis of our previous and current data concerning the influence of immunosuppressive drugs on the disease on the transferred pathologies.

## Methods

### Patients

In total, 11 patients with SSc were enrolled at the Department of Rheumatology, University of Lübeck, Germany. Patients were diagnosed using the 2013 ACR/EULAR classification criteria for SSc [9]. Given that our previous study showed that transfer of PBMC from patients treated with rituximab failed to induce disease in recipient mice [8], in this study, we tried to exclude patients who received strong immunosuppressive drugs, including mycophenolate mofetil, cyclophosphamide, or their combination. All patients agreed to this study by written informed consent. Since we [8] and others [10–12] have convincingly demonstrated that the transfer of PBMC from healthy subjects is not able to induce the production of autoantibodies or tissue inflammation in recipient mice, in the current study, we did not recruit healthy volunteers. This study was performed in accordance with the 1964 Helsinki Declaration, and the approval was obtained from the institutional ethics committee of the University of Lübeck (Number: 16-199, Date: 14/11/2016).

### Mice

Female B10;B6-*Rag2*^*tm1Fwa*^*Il2rg*^*tm1Wjl*^(*Rag2*^*−/−*^*Il2rg*^*−/−*^) mice were purchased from Taconic Biosciences, Inc., USA. These mice were housed under specific pathogen-free conditions with 12-h light/dark cycles at the animal facilities of the Research Center Borstel. All animal studies were approved by the Animal Research Ethics Board of the Ministry of Energy Change, Agriculture, Environment, Nature, and Digitalization, Kiel, Germany (Number: V 241 – 50577/2017 (108-8/17), Date: 05/10/2017).

### Isolation of human PBMC and depletion of human T or B cells

Human PBMC were isolated from the peripheral blood using density gradient medium Pancoll, as previously described [8]. To deplete T or B cells, 2 × 10^7^ human PBMC were incubated with anti-human CD3 microbeads (Miltenyibiotec) or anti-human CD19 microbeads (Miltenyibiotec), respectively, using the AutoMACS program. For the corresponding whole PBMC control samples, 2 × 10^7^ human PBMC without incubation with microbeads went through the AutoMACS program. The eluated PBMC were centrifuged, washed, and resuspened in 100 μl RPMI 1640 medium.

### Adoptive transfer of PBMC

*Rag2*^*−/−*^*Il2rg*^*−/−*^ female mice, 8 to 10 weeks old, were used for the experiments. Then, 2 × 10^7^ PBMC went through the AutoMACS program, resuspended in 100 μl RPMI 1640 medium, and injected intraperitoneally (i.p.) into each recipient mouse. Mice that received whole PBMC, T cell-depleted PBMC, or B cell-depleted PBMC from a patient were regarded as a paired group for comparison.

### Flow cytometry

Frequencies of B cells, CD4^+^ T cells, and CD8^+^ T cells in human PBMC were determined by flow cytometry, as previously described [8]. Briefly, human PBMC with or without T or B cell depletion were stained with anti-human CD3-BV421 (BD Biosciences, clone UCHT1), anti-human CD4-BV650 (BD Biosciences, clone 2RPA-T4), anti-human CD8-APC (BD Biosciences, clone SK1), anti-human CD20-Percp/cy5.5 (BD Biosciences, clone 2H7), and anti-human CD45-FITC (BD Biosciences, clone 2D1) and measured by using LSR II flow cytometer (BD, USA). The data were analyzed using the FACS Express software (De Novo Software, USA, version 5).

### Enzyme-linked immunosorbent assay (ELISA)

Levels of human total IgG, human anti-AT1R, and anti-ETAR abs were determined by using enzyme-linked immunosorbent assays (ELISA) using the method described previously [8]. Human total IgG was determined using a quantitative ELISA, as previously described [8].

### Histology

Murine organs including the skin, lungs, liver, kidneys, muscles, and spleens were collected and fixed in 4% paraformaldehyde for 24 h. After dehydration and paraffinization, the samples were embedded in paraffin and then sectioned at a thickness of 4 μm for histological analysis. For histological evaluation, 4-μm paraffin-embedded sections were stained with hematoxylin and eosin (H&E). The severity of inflammation was defined as the percentage of area with inflammation (affected area/total area ×100%).

### Immunohistochemistry (IHC) staining

Immunohistochemistry (IHC) staining was performed on paraffin sections of mouse spleen sections to detect human CD4^+^ T cells, CD20^+^ B cells, and CD138^+^ plasma cells [5, 7]. After antigen retrieval, endogenous peroxidase, endogenous biotin, and unspecific binding were blocked with a 3% H_2_O_2_ biotin blocking solution (Vector Laboratories, Inc. Burlingame, CA94010, USA) and 5% BSA (Sigma, St. Louis., MO, USA) solution. Subsequently, the sections were incubated overnight at 4 °C with primary antibodies against human CD4 (Abcam, UK, Clone: EPR6855), human CD20 (Dako, Santa Clara, CA, USA, Clone: L26), or human CD138 (Biolegend, San Diego, CA, USA. Clone: M15), followed by incubation with biotin-conjugated goat anti-rabbit IgG (Jackson ImmunoResearch Laboratories. Inc., Baltimore, USA) or goat anti-mouse IgG (Jackson ImmunoResearch Laboratories. Inc., Baltimore, USA). Finally, the immunoreactivity was visualized with 3,3-diaminobenzadine (DAB substrate kit, Vector Laboratories, Inc. Burlingame), and the sections were counterstained with hematoxylin.

### Statistical analysis

Data were analyzed using the GraphPad5 Prism software (La Jolla, CA, USA). Quantitative data with normal distribution are presented as mean ± standard deviation (mean ± SD), while data without normal distribution are expressed as medians (min, max). To assess the significance of the differences in quantitative data, the paired *t* test was applied when values were under normal distribution, while the Wilcoxon matched pairs test was performed when values were not normally distributed. Differences in the rates of mouse death were analyzed by using Fisher’s exact test. The cumulative survival curve was analyzed by using Kaplan-Meier analysis. Since most variables analyzed were not normally distributed, Spearman correlation was applied for the analysis for the evaluation of quantitative variables. For evaluating the effect of treatment of patients with chemical immunosuppressive drugs on the pathogenicity of PBMC in this humanized mouse model, mice that received PBMC from SSc patients who were treated with various chemical immunosuppressive drugs were compared with those that received PBMC from patients without treatment of any immunosuppressive drugs. A *p* value < 0.05 was considered statistically significant.

## Results

### Demographic and clinical features of patients with SSc

In total, 11 SSc patients (3 males and 8 females) with 9 lcSSc patients and 2 dcSSc were enrolled in this study. The demographic and clinical characteristics of these patients are summarized in Supplementary Table 1. The mean age and median disease duration of SSc patients were 57 years and 5.1 years, respectively. Of the 11 patients, 9 had positive ANA, 4 had positive anti-Scl70 abs, and 4 had positive ACA. All 11 patients developed Raynaud syndrome, and part of them showed complications in the lungs, kidneys, heart, and *gastrointestinal* tract. Supplementary Table 1 also summarizes the treatment information of those patients.

### Depletion of T or B cells from human PBMC

PBMC were isolated from 11 patients with SSc, with a cell viability of more than 90% which was indicated by the trypan blue exclusion test. After the depletion of T or B cells, the depletion efficiency was evaluated by using FACS analysis. As shown in Suppl. Fig. 1, T cell depletion resulted in a dramatic and significant decrease in human CD3^+^ T cells in PBMC (from 65.7 ± 11.9% to 10.2 ± 11.9%). Further evaluation of the two major subtypes of T cells demonstrated that CD4^+^ T cells were almost completely depleted in all samples (from 39.3 ± 10.9% to 1.81 ± 1.45%), while CD8^+^ T cells could only partially be removed (from 21.2 ± 5.55% to 6.55 ± 9.15%). Ablation of B cells was highly efficient, and a complete deficiency of CD20^+^ B cells was observed in all samples (from 7.46 ± 4.01% to 0.08 ± 0.05%).

Then, 2 × 10^7^ PBMC, with or without depletion of T or B cells, were transferred into *Rag2*^*−/−*^*/Il2rg*^*−/−*^ mice via i.p. injection. Eighteen mice were transferred with whole PBMC (*n* = 6), T cell-depleted PBMC (*n* = 6), or B cell-depleted PBMC (*n* = 6) from 6 patients with SSc based on the available cell number. Two mice were transferred with whole PBMC (*n* = 1) or T cell-depleted PBMC (*n* = 1) from one SSc patient. Eight mice were transferred with whole PBMC (*n* = 4) or B cell-depleted PBMC (*n* = 4) from 4 SSc patients. Taken together, this experiment consisted of 7 paired groups of whole PBMC vs T cell-depleted PBMC and 10 paired groups of whole PBMC vs B cell-depleted PBMC. Six weeks after the transfer, human leukocytes could be detected in the peripheral blood of the recipient mice of all three groups. As compared with mice that received whole PBMC, mice that received T cell-depleted PBMC, but not those that received B cell-depleted PBMC, showed significantly lower levels of circulating human leukocytes (Suppl. Fig. 2).

### Depletion of T or B cells inhibits PBMC transfer-induced death

**Fig. 1:**
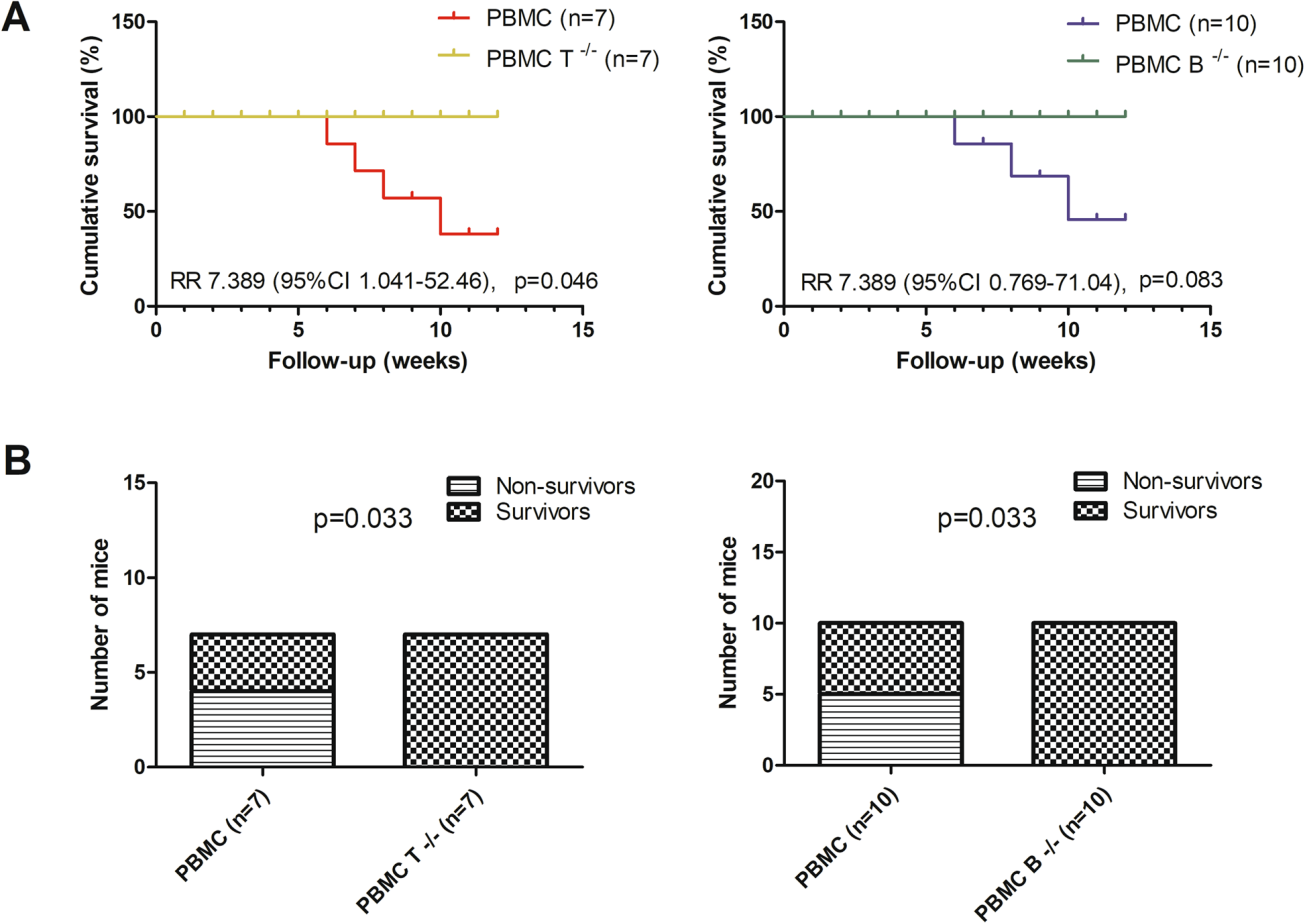
Mortality in mice after engraftment with patient-derived PBMC. **A.** Kaplan-Meier survival curve illustrating the survival difference between whole PBMC-transferred mice and mice transferred with T or B cell-depleted PBMC. **B.** Mice transferred with whole PBMC showed significantly higher death rates compared to mice transferred with T or B cell-depleted PBMC. Comparison of survival curves (**A**) and mortality rates (**B**) between whole PBMC-transferred mice and mice transferred with T or B cell-depleted PBMC was determined using the log-rank (Mantel-Cox) test and Fisher’s exact test, respectively. *p* values reflect the comparisons between mice transferred with whole PBMC and mice transferred with T or B cell-depleted PBMC. PBMC, mice transferred with whole PBMC from SSc patients, PBMC T^-/-^, mice transferred with T cell-depleted PBMC from SSc patients, PBMC B^-/-^, mice transferred with B cell-depleted PBMC from SSc patients

Six mice transferred with whole PBMC from patients with SSc died or had to be sacrificed due to *body weight reduction* of 20% or *more* weight before the end of the experiment, while such death was not observed in mice transferred with T or B cell-depleted PBMC from SSc patients. Kaplan-Meier analysis showed a significant difference in cumulative survival between the whole PBMC-transferred group and T cell-depleted PBMC-transferred group as well as between the PBMC-transferred group and B cell-depleted PBMC-transferred group (Fig. 1A). Accordingly, whole PBMC-transferred mice had significantly higher mortality rates than mice transferred with T or B cell-depleted PBMC (Fig. 1B).

## Depletion of T or B cells prevents the restoration of white pulp in the murine spleen

**Fig. 2:**
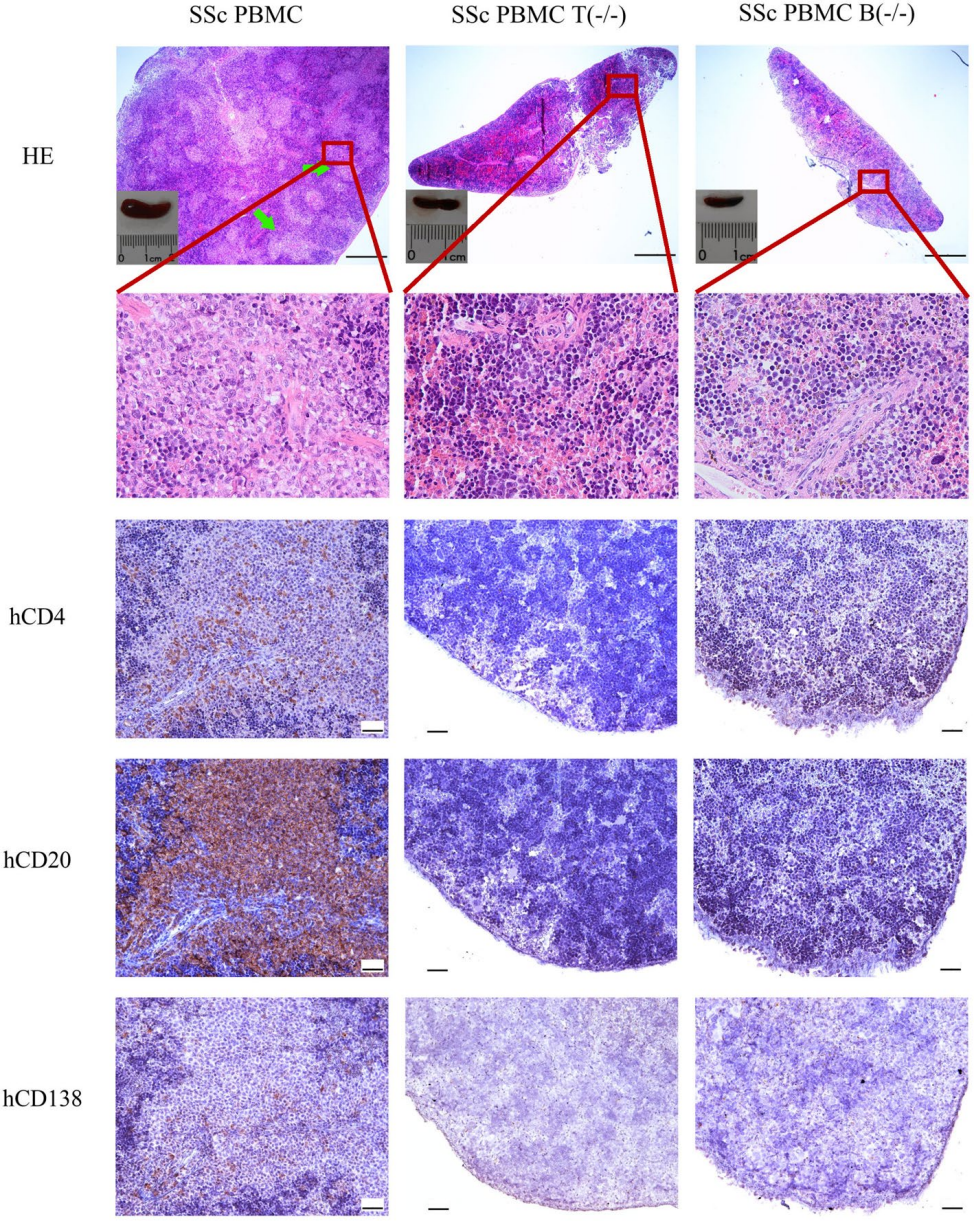
Morphology of the splenic white pulp and human lymphocytes in the spleens of recipient mice. Representative micrographs of H&E-stained spleen sections and IHC-stained spleen sections for detecting human CD4^+^ T, CD20^+^ B, and CD138^+^ plasma cells. Green arrows indicate white pulps. PBMC, mice transferred with whole PBMC from SSc patients; PBMC T(-/-), mice transferred with T cell-depleted PBMC from SSc patients; PBMC B(-/-), mice transferred with B cell-depleted PBMC from SSc patients. Bars indicate 500 µm and 50 μm in HE-stained spleen sections and IHC-stained spleen sections, respectively

Previously, we demonstrated that the transfer of PBMC from patients with SSc restores the splenic white pulp in immunodeficient mice [5]. Next, we determined the effect of depletion of T or B cells on this restoration. As expected, the spleens of *Rag2*^*−/−*^*Il2R*^*−/−*^ mice transferred with whole PBMC from SSc patients were characterized by well-structured white pulps (Fig. 2). However, such well-structured white pulps were not observed in the spleens of mice transferred with T cell-depleted PBMC (0 out of 7) and were rarely observed in the spleens of mice transferred with B cell-depleted PBMC (2 out of 10, Fig. 2).

The distribution of human lymphocytes in the murine spleen was investigated using IHC staining. As shown in Fig. 2, the well-structured splenic white pulps in mice transferred with whole PBMC from SSc patients consisted predominantly of human CD20^+^ B cells and CD4^+^ T cells as well as a small amount of human CD138^+^ plasma cells. However, very few CD4^+^ T cells, CD20^+^ B cells, or CD138^+^ plasma cells could be detected in the spleens of all the mice transferred with T cell-depleted PBMC. With regard to mice transferred with B cell-depleted PBMC, a small amount of CD4^+^ T cells, but neither CD20^+^ B cells nor CD138^+^ plasma cells were observed in the spleen without white pulp (Fig. 2), and many T cells but not B cell or plasma cells could be detected in the murine spleen with white pulp-like structure (data not shown).

### Depletion of T or B cells decreases the production of human IgG and autoantibodies

**Fig. 3:**
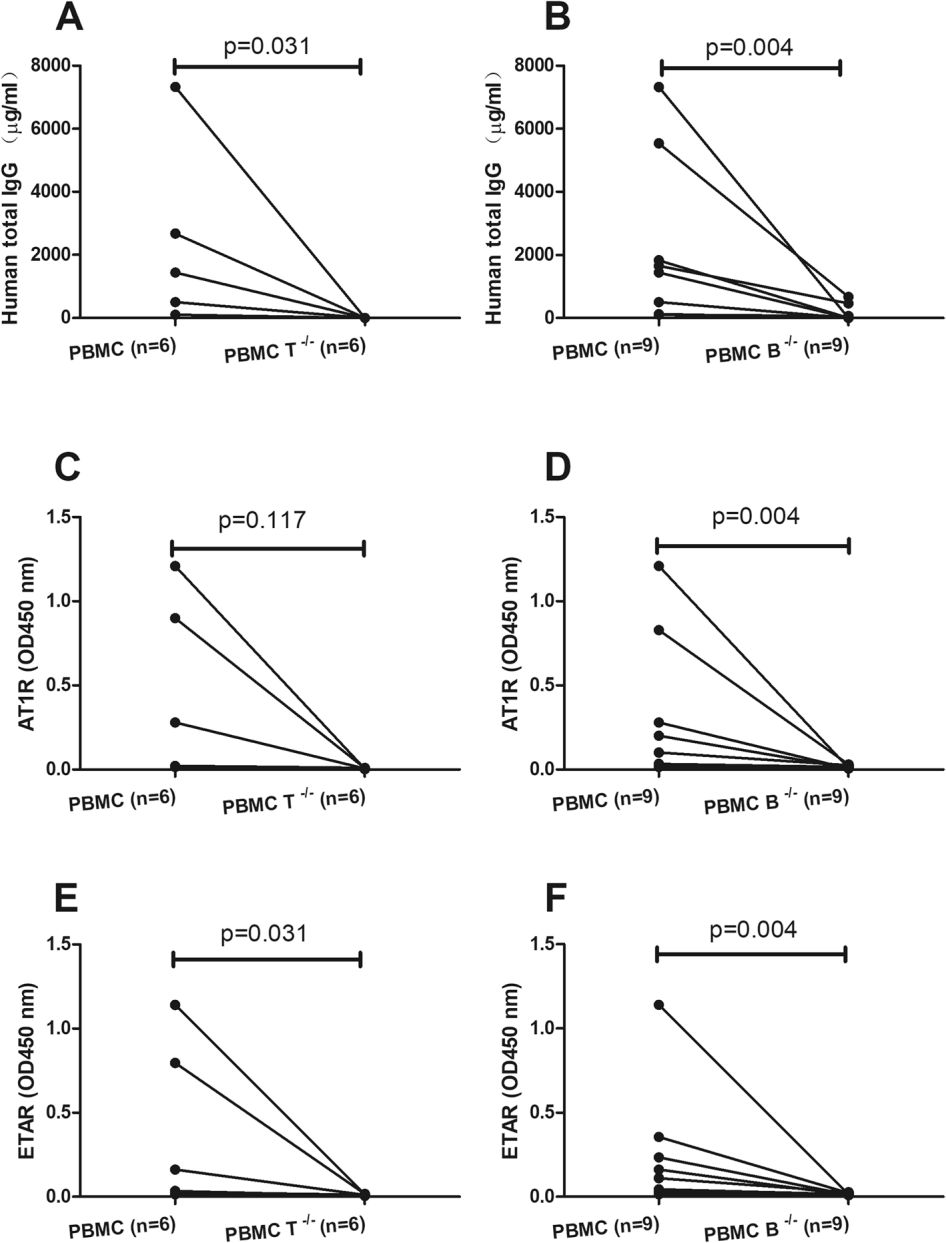
Production of human antibodies in recipient mice. Comparison of serum levels of total human IgG (**A**, **B**), anti-AT1R IgG (**C**, **D**), and anti-ETAR IgG (**E**, **F**) between mice transferred with whole PBMC from SSc patients and mice transferred with T cell-depleted (*n* = 6) or B cell-depleted (*n* = 9) PBMC. *p* values reflect the comparisons between mice transferred with whole PBMC and mice transferred with T or B cell-depleted PBMC. Statistical significance of comparison was determined using the Wilcoxon matched pairs test, and statistical significance of comparison in **C** was determined by the paired *t* test

Given the depletion of T or B cells affects the restoration of white pulp and the presence of human plasma cells in the murine spleen, we next determined the human IgG and abs in the murine sera. For the 6 mice that died or had to be sacrificed before the end of the experiment, the serum samples of 5 of them were collected when they died, and serum sample from one mouse was not available. The sera from all other mice were prepared at week 12 after the transfer. As shown in Fig. 3A, B, mice transferred with whole PBMC from SSc patients generated a considerable amount of human IgG. When compared to mice transferred with whole PBMC, both mice transferred with T cell-depleted PBMC (*p* = 0.031) and mice transferred with B cell-depleted PBMC (*p* = 0.004) produced dramatically and significantly less amount of human IgG. In addition, we also determined levels of anti-AT1R IgG and anti-ETAR IgG which have been shown to be associated with susceptibility, severity, and mortality in SSc [13–15]. Mice transferred with whole PBMC from SSc patients produced both anti-AT1R and anti-ETAR IgG abs which were neither detected in mice transferred with T cell- nor B cell-depleted PBMC (Fig. 3C–F). According to our previous study, approximately 30% of mice that received PBMC from SSc patients produced anti-nuclear antibodies (ANA), and ANA patterns were consistent with those observed in the corresponding SSc donors [8]. Next, we determined whether depletion of T or B cells affects the production of ANA in recipient mice. ANA were detected in the sera of 2 out of 7 mice transferred with patient-derived PBMC. Moreover, in line with previous results, ANA patterns of both positive sera were consistent with those of the two corresponding patients. By contrast, mice that received T cell-depleted or B cell-depleted PBMC isolated from the 2 SSc patients scored ANA negative (Suppl. Fig. 3).

### Tissue inflammation in recipient mice after transfer of human PBMC

To assess the effect of human T and B cells in disease manifestations, the histopathology of mouse tissues including the lungs, kidneys, liver, heart, muscles, intestines, esophagus, and skin was evaluated. Although 6 mice that received whole PBMC from SSc patients died or were sacrificed before the end of the experiment, the tissues of all those mice were collected and analyzed.

**Fig. 4:**
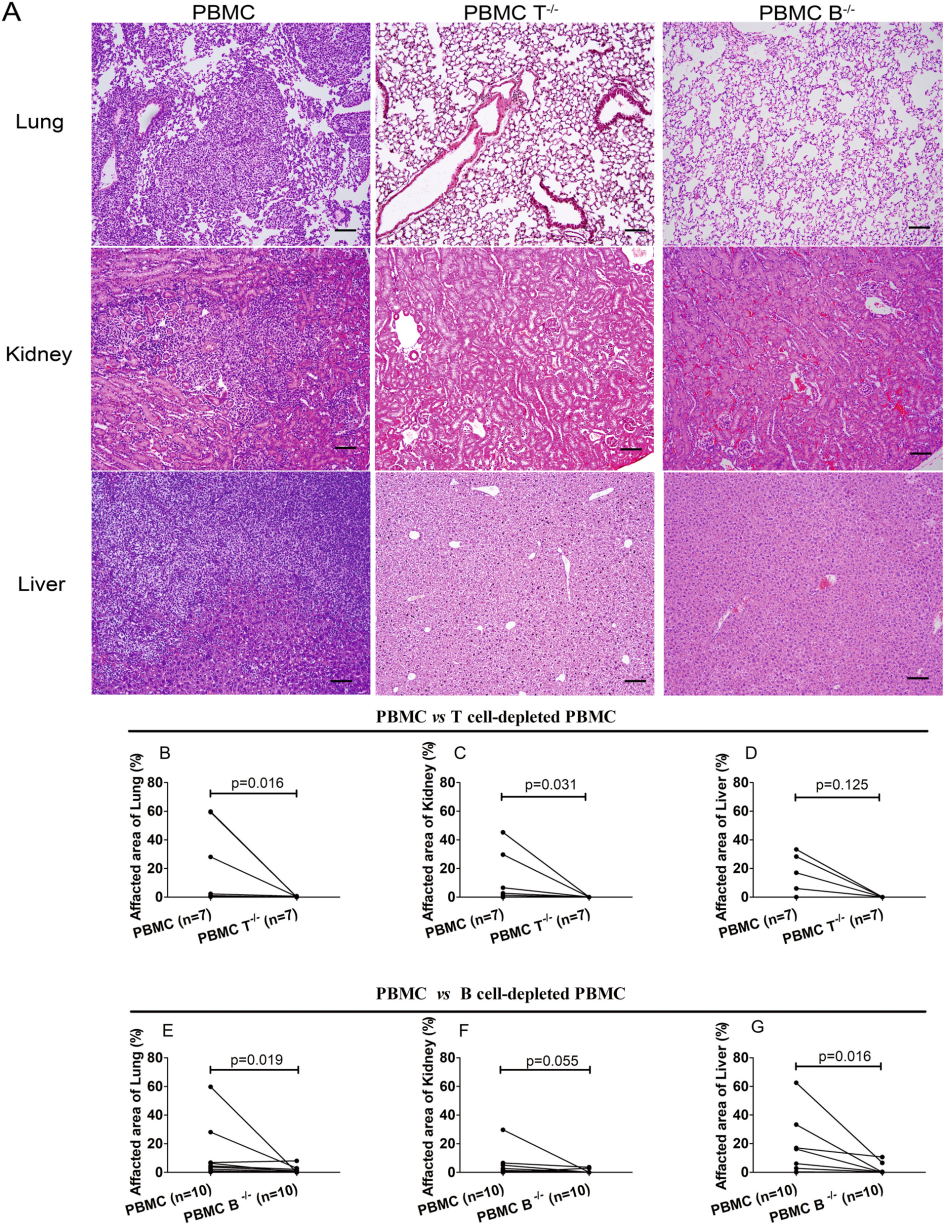
Histopathology of the lungs, kidneys, and liver of recipient mice. **A** Representative micrographs of H&E-stained paraffin-embedded section of the lungs, kidneys, and liver of mice transferred with whole PBMC, T cell-depleted PBMC, or B cell-depleted PBMC from patients with SSc. Bars = 100 μm. **B**–**D** Severity of inflammation in the lungs, kidneys, and liver in mice transferred with whole PBMC and mice transferred with T cell-depleted PBMC from SSc patients. **E**–**G** Severity of inflammation in the lungs, kidneys, and liver between mice transferred with PBMC and mice transferred with B cell-depleted PBMC from SSc patients. Severity of inflammation was calculated as the percentage of areas affected by the infiltrated inflammation. Statistical significance was determined by Wilcoxon matched pairs test, and *p* values are indicated

Mice transferred with whole PBMC from SSc patients showed inflammation in the lungs, kidneys, and liver (Fig. 4), but not in the heart, muscles, intestines, esophagus, or skin (data not shown). However, little or no inflammation in the lungs, kidneys, and liver was observed in mice transferred with T cell-depleted PBMC or B cell-depleted PBMC (Fig. 4). Therefore, these results suggest that both T and B cells are required for the disease manifestation in this humanized mouse model for SSc.

To clarify the relationship between tissue inflammation, circulating human leukocytes, and antibodies in the murine peripheral blood, we performed a correlation analysis with 10 mice that received whole PBMC. The severity of inflammation in the lungs, kidneys, and liver, as well as levels of circulating human CD45^+^ leukocytes, CD3^+^ T cells, CD4^+^ T cells, CD8^+^ T cells, and CD20^+^ B cells at the 6th week after the cell transfer, and levels of total IgG, anti-AT1R IgG, and anti-ETAR IgG in the sera obtained after the sacrifice of mice were included in this analysis. As shown in Supplementary Fig. 4, lung inflammation was significantly correlated with the levels of circulating human CD20^+^ B cells, anti-AT1R IgG, and anti-ETAR IgG.

### Effect of immune suppressive drugs in patients on the PBMC transfer-induced disease manifestation in mice

**Fig. 5:**
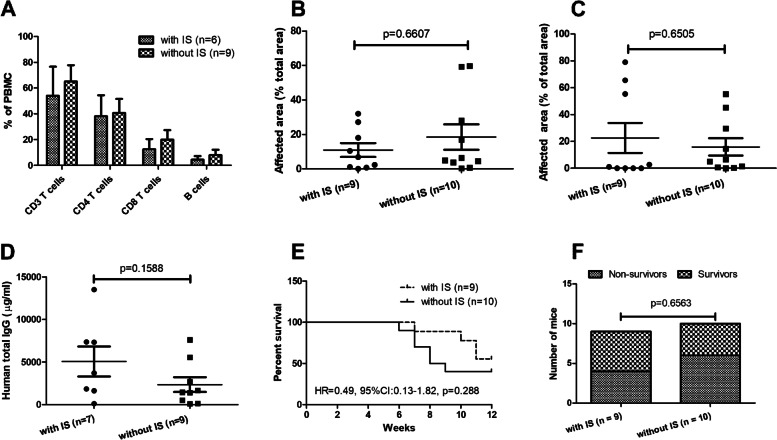
Effect of immunosuppressive therapies in patients on disease manifestations in mice. Relative amounts of various lymphocyte subsets in PBMC of SSc patients treated with or without immunosuppressive (IS) drugs. The percentages of CD3^+^, CD4^+^, and CD8^+^ T cells as well as of CD20^+^ B cells in freshly isolated PBMC were detected by FACS before transfer into recipient mice (**A**). Comparison of severity of inflammation in the lungs (**B**) and kidneys (**C**), human IgG (**D**), survival curves (**E**), and mortality rates (**F**) between mice that received PBMC from patients treated with immunosuppressive (IS) drugs and those that received PBMC from patients without IS drugs. *p* values for comparisons in **A**–**D** were calculated using the Mann *U* test, and those indicated in **E** and **F** were calculated using the log-rank (Mantel-Cox) test and Fisher’s exact test, respectively

Chemical immune suppressive drugs widely used in SSc treatment are suspected to affect lymphocytes [16] Given the indispensable role of human T and B cells in this humanized mouse model, we next investigated whether treatment of patients with such drugs affects the PBMC transfer-induced disease manifestation in recipient mice. For this purpose, we performed an analysis with the data from the current study and our previous study [8]. In total, PBMC from 7 SSc patients who were treated with various chemical immunosuppressive drugs such as mycophenolate mofetil, prednisolone, methotrexate, azathioprine, or ciclosporin were transferred to 9 recipient mice, while PBMC from 9 SSc patients without treatment of any immunosuppressive drugs were transferred into 10 recipient mice (Supplementary Table 2). We first evaluated the percentages of CD3^+^, CD4^+^, and CD8^+^ T cells as well as CD20^+^ B cells in PBMC isolated from patients with SSc. As shown in Fig. 5A, although the mean values of all above four subsets of lymphocytes were slightly lower in PBMC from patients treated with immunosuppressive drugs than those from patients without treatment of immunosuppressive drugs, none of the differences was statistically significant.

After the transfer, mice that received PBMC from immunosuppressive drug-treated patients showed similar levels of inflammation in the lungs and kidneys as those that received PBMC from patients without treatment of immunosuppressive drugs (Fig. 5B, C). Moreover, the two groups of mice produced comparable levels of human IgG (Fig. 5D). Within 12 weeks after the transfer of PBMC from patients, 4 out of 9 mice from the group with immunosuppressive drugs and 6 out of 10 mice from the group without immunosuppressive drugs died or had to be sacrificed. The mortality rate was not significantly different between the two groups (Fig. 5E, F).

## Discussion

In the present study, we investigated the role of human T and B cells in a humanized mouse model of SSc in which disease features are induced by the adoptive transfer of patient-derived PBMC into *Rag2*^*−/−*^*Il2rg*^*−/−*^ mice. Consistent with our previous findings [8], the transfer of whole PBMC from SSc patients could restore the well-structured splenic white pulps and induce the production of human IgG and SSc-related abs as well as the development of systemic inflammation in recipient mice. However, when those PBMC were in vitro depleted from T or B cells before the transfer, recipient mice did not show any of those immunological, histopathological, or clinical features.

By in vitro depleting B cells before the transfer, we confirmed the indispensable role of B cells in the humanized mouse model [8]. These findings are in line with the clinical observations that B cell activation is associated with the development and progression of SSc [17, 18]. Moreover, this finding is also supportive for the treatment of SSc patients with B cell-targeting therapeutics. Currently, rituximab has been used in the treatment of SSc, especially in its diffused cutaneous form [19–23], and it has been shown that rituximab is well tolerated and shows beneficial effects in skin sclerosis, joint involvement, and pulmonary fibrosis [24, 25]. As antibody-producing cells, B lymphocytes might contribute to the development of disease manifestations by generating antibodies. Besides autoantibody production, B cells are capable to contribute to the disease development in SSc or its animal models by releasing cytokines and acting as antigen-presenting cells. For example, it has been suggested that B cell-derived IL-6 contribute to the disease manifestation in the tight-skin mouse [26]. In addition, B cells could act as APCs and thus influence the function of T cells [27].

Germinal centers (GCs) are specialized compartments in secondary lymphoid organs, and they are vital for the development of effective adaptive immune responses [28]. In the current study, immunodeficient mice transferred with whole PBMC from SSc patients developed splenic white pulp which is composed of CD4 T cells, B cells, and plasma cells, suggesting the formation of GCs. By contrast, the splenic white pulp was not observed in mice that received T cell-depleted PBMC. In addition, although two out of 10 mice transferred with human B cell-depleted PBMC formed splenic white pulp-like structures, the spleens did not contain B or plasma cells. Accordingly, these two mice did not produce abs, suggesting that these white pulp-like structures contain no functional GCs. Therefore, these results support that both T and B cells are indispensable for the formation of functional GCs, which is in line with the notion that both B and T cells are fundamental components of GCs [29]. These findings also indicate an essential role in the interaction between T cells and B cells in the formation of GC, and further investigations on the regulation of this process will be of interest.

Interestingly, recipient mice transferred with whole PBMC from SSc patients generated autoantibodies against AT1R and ETAR, while mice transferred with B cell- or T cell-depleted PBMC were unable to produce these antibodies, suggesting that the two abs are produced in a T cell-dependent manner. Previously, Riemekasten and colleagues have demonstrated that these two abs are associated with severe disease manifestations and mortality. Moreover, in vitro studies have revealed that anti-AT1R and anti-ETAR abs are functional abs with agonistic effects on their receptor [13–15], suggesting that anti-AT1R and anti-ETAR IgG are SSc-associated abs and potential contributors to the pathogenesis of the disease. The current study demonstrated that B cells are indispensable for the disease manifestations in this humanized mouse model, indicating that autoantibodies might play an essential role in this experimental model too. Moreover, a positive correlation was observed between severity of lung inflammation and levels of anti-AT1R IgG and anti-ETAR IgG, supporting a role of those autoantibodies in the development of this manifestation in this humanized mouse model. However, since SSc is featured by multiple autoantibodies, the contribution of anti-AT1R and anti-ETAR IgG abs in this model needs to be validated experimentally in future studies.

The current study demonstrated that PBMC from patients treated with chemical immunosuppressive drugs show comparable pathogenicity as those from patients without treatment with immunosuppressive drugs, indicating that their protective effect in patients is not transferrable into the mice. This is in contrast to our previous observation that PBMC isolated from SSc patients treated with rituximab show little pathogenicity in recipient mice and is, most likely, a direct consequence of the different therapeutic principles of both approaches. While treatment with rituximab represents a cell-targeting therapy resulting in a long-lasting depletion of B cells, immunosuppressive drugs mainly target the function and proliferation of lymphocytes as well as the expression of inflammatory mediators, which is reversible after the discontinuation of the drug. Therefore, the capacity to transfer the disease from patients in experimental therapy to mice may represent a novel parameter to estimate the efficacy and sustainability of novel therapeutic concepts. However, it needs to be stated that the immunosuppressive medication in our patient samples represents a heterogenous group of chemical drugs, and patients treated with strong immunosuppressive drugs such as mycophenolate mofetil and cyclophosphamide were largely excluded from the study. Therefore, the finding does not exclude the possibility that treatment with a specific immunosuppressive drug, particularly those with a strong immunosuppressive effect, might reduce the pathogenicity of PBMC in SSc patients. In addition, due to the limited number of patients and mice analyzed in this study, these findings need to be interpreted carefully and should be further validated with more samples.

Previous studies have shown that transfer of PBMC from patients with primary biliary cirrhosis [30], systemic lupus erythematosus [10], and myasthenia gravis [12] induces tissue-specific inflammation in the liver, kidneys, and muscle, respectively, in recipient mice. By contrast, we could show that the transfer of PBMC from patients with SSc induced systemic inflammation in multiple organs [8]. The difference in tissue involvement between mouse models of autoimmune disorders suggests that disease manifestation in recipient mice is disease-specific. Interestingly, in the current study, mice developed systemic inflammation in the lungs, kidneys, and liver, but no signs of myositis after PBMC transfer from SSc patients, which was different to our previous study [5]. In the latter, 4 out of 6 SSc PBMC donors suffered from myalgias or myositis [5], while these manifestations were not present in the 11 patients recruited for this study. This finding supports our previous hypothesis that the humanized mouse model reflects, at least partially, the organ involvement of their corresponding patient donors.

Although the humanized mouse model provides a novel tool for investigating the pathogenesis of SSc, two major limitations of the model need to be mentioned here. First, SSc is a disease featured by autoimmunity, inflammation, fibrosis, and vasculopathy, while the humanized model used in this study only mimics parts of the disease features, namely autoimmunity and systemic inflammation, representing the early phase of SSc. Therefore, this model is not suitable for investigating the pathogenesis of fibrosis and vasculopathy. Second, transfer studies are ideally executed with PBMC of patients without any treatment. However, due to the limited numbers of SSC patients available, we had to recruit patients in this study which were under therapy with various drugs. We cannot exclude that some treatments might affect the outcome of the PBMC-induced pathology in mice.

In conclusion, this study provides, for the first time, direct evidence that both human T and B cells play a crucial role in the humanized mouse model for SSc.

## Supplementary Information

**Additional file 1: Supplementary Table 1.** Demographic and clinical features of SSc patients recruited for the in vitro T or B cell-depletion. **Supplementary Table 2.** Overview on medications given to all individual SSc patients with SSc treated with or without immunosuppressive drugs in the present and previous study [5]. **Supplementary Figure 1.** Efficiency of depletion of human T or B cells from PBMC. Levels of CD3+ T cells (A), CD4+ T cells (B), CD8+ T cells (C), and CD20+ B cells (D) were determined by FACS analysis and presented as % of total human leukocytes in PBMC. Statistical significance of comparison was determined using the paired t test. **Supplementary Figure 2.** Levels of human leukocytes in peripheral blood of recipient mice. Mice were transferred with whole PBMC, T-cell depleted, or B-cell depleted PBMC, and peripheral blood was taken at the 6^th^ week (A, B) and 12^th^ week (C, D) after the transfer. Subsequently, human CD45^+^ leukocytes, CD3^+^ T cells, CD4^+^ T cells, CD8^+^ T cells and CD20^+^ B cells were identified by flow cytometry. Levels of human leukocytes are presented as percentage of total leukocytes including murine and human leukocytes in murine blood. Comparison on levels of human leukocytes indicated between mice transferred with whole PBMC (*n* = 7) and mice transferred with T-cell depleted PBMC (*n* = 7) (A) and between mice transferred with whole PBMC (*n* = 10) and mice transferred with B-cell depleted PBMC (*n* = 10) (B) by week by the 6^th^ week after the transfer. Comparison on levels of human leukocytes indicated between mice transferred with whole PBMC (*n* = 4) and mice transferred with T-cell depleted PBMC (*n* = 4) (C) and between mice transferred with whole PBMC (*n* = 16) and mice transferred with B-cell depleted PBMC (*n* = 6) (D) by week by the 12^th^ week after the transfer. P values reflect comparisons between mice transferred with whole PBMC and mice transferred with T- or B-cell depleted PBMC. Statistical significance was determined using the Wilcoxon matched pairs test. **Supplementary Figure 3.** Presence of ANA in sera of PBMC-transferred mice. Antinuclear antibody (ANA) pattern of murine sera were detected using (HEp-2) cell-based immunofluorescence staining (EUROPattern Suite, Euroimmun, Germany). Two out of 7 mice which received whole PBMC from SSc patients scored positive for ANA, and their ANA patterns were consistent with those of two corresponding SSc patients. By contrast, mice which received T cell-depleted or B cell-depleted PBMC isolated from the 2 SSc patients were ANA negative. Representative micrographs of the ANA test for mice received whole PBMC, T cell-depleted PBMC (PBMC T^-/-^) or B cell-depleted PBMC (PBMC B^-/-^) are shown. **Supplementary Figure 4.** Matrix of Spearman’s correlation coefficients between immunological and histopathological variables. Severity of inflammation in the lung, kidney and liver as well as levels of circulating human CD45^+^ leukocytes, CD3^+^ T cells, CD4^+^ T cells, CD8^+^ T cells and CD20^+^ B cells at 6^th^ week after the cell transfer, and levels of total IgG, anti-AT1R IgG and anti-ETAR IgG in sera obtained after the sacrifice of mice were used for the analysis. Samples were tested for normal distribution by using Shapiro-Wilk normality test. Since most variables were not normally distributed, Spearman correlation was applied for the analysis. A color-coded correlation scale is provided on the right of the plot. Blue and ellipses represent negative and positive correlations, respectively, and darker color tones representd larger correlation coefficient magnitudes. Statistically significant differences are indicated by asterisks (*p<0.05, **p<0.01 and ***p<0.001).

## Data Availability

The original contributions presented in the study are included in the article and supplementary material. Further inquiries can be directed to the corresponding authors.

## Abbreviations

PBMC: Peripheral blood mononuclear cells
SSc: Systemic sclerosis
AT1R: Angiotensin-II type 1 receptor
ETAR: Endothelin-1 type A receptor
ELISA: Enzyme-linked immunosorbent assays
H&E: Hematoxylin and eosin
IHC: Immunohistochemistry
