# Supplementary material for: Both T and B cells are indispensable for the development of a PBMC transfer-induced humanized mouse model for SSc
Source: Arthritis Res Ther. 2022 Aug 25;24:209. doi: 10.1186/s13075-022-02896-6 (PMC9404611; doi:10.1186/s13075-022-02896-6)
Supplement: Supplementary file 1 — Additional file 1: Supplementary Table 1. Demographic and clinical features of SSc patients recruited for the in vitro T or B cell-depletion. Supplementary Table 2. Overview on medications given to all individual SSc patients with SSc treated with or without immunosuppressive drugs in the present and previous study [5]. Supplementary Figure 1. Efficiency of depletion of human T or B cells from PBMC. Levels of CD3+ T cells (A), CD4+ T cells (B), CD8+ T cells (C), and CD20+ B cells (D) were determined by FACS analysis and presented as % of total human leukocytes in PBMC. Statistical significance of comparison was determined using the paired t test. Supplementary Figure 2. Levels of human leukocytes in peripheral blood of recipient mice. Mice were transferred with whole PBMC, T-cell depleted, or B-cell depleted PBMC, and peripheral blood was taken at the 6th week (A, B) and 12th week (C, D) after the transfer. Subsequently, human CD45+ leukocytes, CD3+ T cells, CD4+ T cells, CD8+ T cells and CD20+ B cells were identified by flow cytometry. Levels of human leukocytes are presented as percentage of total leukocytes including murine and human leukocytes in murine blood. Comparison on levels of human leukocytes indicated between mice transferred with whole PBMC (n = 7) and mice transferred with T-cell depleted PBMC (n = 7) (A) and between mice transferred with whole PBMC (n = 10) and mice transferred with B-cell depleted PBMC (n = 10) (B) by week by the 6th week after the transfer. Comparison on levels of human leukocytes indicated between mice transferred with whole PBMC (n = 4) and mice transferred with T-cell depleted PBMC (n = 4) (C) and between mice transferred with whole PBMC (n = 16) and mice transferred with B-cell depleted PBMC (n = 6) (D) by week by the 12th week after the transfer. P values reflect comparisons between mice transferred with whole PBMC and mice transferred with T- or B-cell depleted PBMC. Statistical significance was determined using the Wilcoxon [file 13075_2022_2896_MOESM1_ESM.docx]

**Supplementary information**

**Supplementary table 1. Demographic and clinical features of SSc patients recruited for the *in vitro* T or B cell-depletion**

|  | Patients with SSc (n = 11) |
| --- | --- |
| **Age (years, mean** ± **SD)** | 57.0 ± 13.8 |
| **Gender (female, n, %)** | 8 (72.7) |
| **Disease duration (years, median (min, max)** | 5.1 (1-16) |
| **SSc classification** (n, %) |  |
| lcSSC | 9 (81.8) |
| dcSSc | 2 (18.2) |
| **Antibody positivity** (n, %) |  |
| ANA | 9 (81.8) |
| Anit-Scl70 antibodies | 4 (36.4) |
| ACA | 2 (18.2) |
| AMA-M2 | 2 (18.2) |
| Anti-Th/To antibodies | 1 (9.1) |
| anti-NOR-90 antibodies | 1 (9.1) |
| Anti- RNA-polymeraseIII antibodies | 1 (9.1) |
| Anti-AT1R antibodies | 4 (36.4) |
| Anti-ETAR antibodies | 3 (27.3) |
|  |  |
| **Symptoms (n, %)** |  |
| Raynaud-syndrome | 11 (100) |
| Fatigue | 3 (27.3) |
| Lung fibrosis | 4 (36.4) |
| PAH | 3 (27.3) |
| Reduced GFR and/or proteinuria (Stix ≥ 1+) | 4 (36.4) |
| Digital ulcers/pits  Cardiac involvement | 8 (72.7)  3 (27.3) |
| Calcinosis, ulcera | 4 (36.4) |
| Abnormal nailfold capillaries | 4 (36.4) |
| Arthralgia | 1 (9.1) |
| Sicca-symptom | 3 (27.3) |
| Gastroesophageal reflux | 3 (27.3) |
| **Treatment (n, %)** |  |
| prednisolone  azathioprine | 3 (27.3)  1 (9.1) |
| mycophenolat mofetil | 1 (9.1) |
| cyclosporine  sulfasalazine*  methotrexate | 1 (9.1)  1 (9.1)  1 (9.1) |
| hydroxychloroquine | 6 (54.4) |
| etoricoxib | 1 (9.1) |
| bosentan | 3 (27.3) |
| betablockers | 1 (9.1) |
| ACE inhibitors | 3 (27.3) |
| Calcium channel blockers (CCB) | 1 (9.1) |
| iloprost i.v. . | 1. (90.9) |
|  |  |

*the treatment of the patient with sulfasalazine stopped 4 weeks before the donation of the PBMC. SSc, systemic sclerosis; lcSSc, limited cutaneous SSc; dcSSc, diffuse cutaneous SSc; ANA, anti-nuclear antibodies; ACA, anti-centromere-specific antibodies; AMA, anti-mitochondrial antibody; anti-NOR-90, anti- a 90-kDa component of the nucleolus-organizing region of chromatin. AT1R, angiotensin-II type1 receptor; ETAR, endothelin-1 type A receptor; PAH, pulmonary arterial hypertension.

**Supplementary table 2. Overview on medications given to all individual SSc patients with SSc treated with or without immunosuppressive drugs in the present and previous study (5).**

|  | **Patient ID** | **Treatment** | **Number of recipient mice** |
| --- | --- | --- | --- |
| **Patients treated with immunosuppresive drugs** | **1** | mycofenolat mofetil, prednisone, cyclosporine, iloprost | **1** |
|  | **2** | prednisone, hydroxychloroquine, iloprost | **1** |
|  | **3** | azathioprine, ACE inhibitor, betablocker, iloprost | **1** |
|  | **4** | methotrexate, iloprost | **2** |
|  | **5** | metothrexate, prednisone, hydroxychloroquine | **1** |
|  | **6** | prednisone, hydroxychloroquine, bosentan. | **2** |
|  | **7** | iloprost; sulfasalazine*, ACE inhibitor | **1** |
| **Patients treated without immunosuppressive drugs or immunomodulators** | **8** | ACE inhibitors, iloprost | **1** |
|  | **9** | hydroxychloroquine; etoricoxib, iloprost | **1** |
|  | **10** | iloprost; bosentan | **1** |
|  | **11** | iloprost | **1** |
|  | **12** | iloprost; CCB, bosentan, hydroxychloroquine | **1** |
|  | **13** | None | **1** |
|  | **14** | hydroxychloroquine | **1** |
|  | **15** | betablocker, ACE inhibitor, | **1** |
|  | **16** | hydroxychloroquine; iloprost | **2** |

*the treatment of the patient with sulfasalazine stopped 4 weeks before the donation of the PBMC.


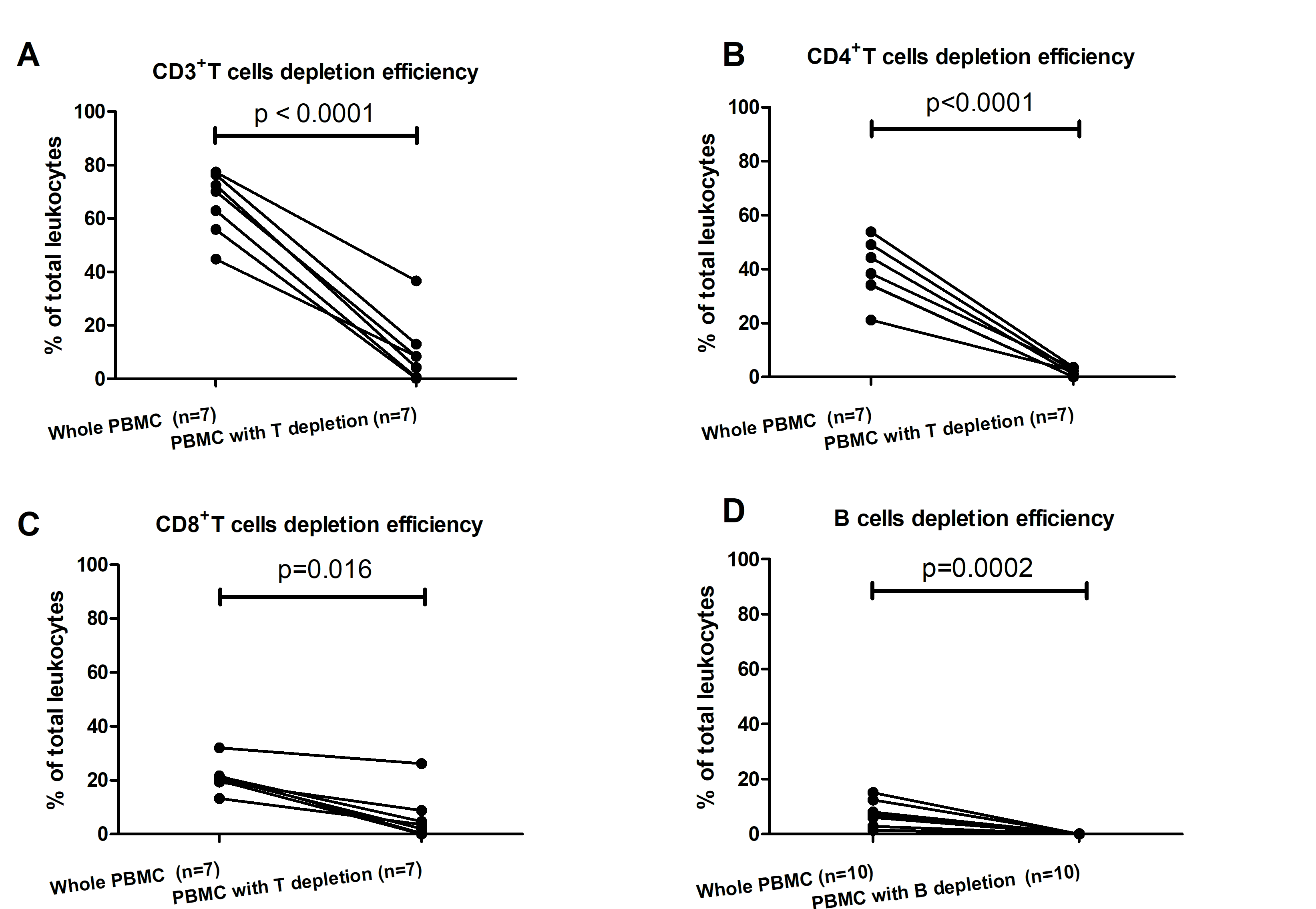


**Supplementary figure 1. Efficiency of depletion of human T or B cells from PBMC.** Levels of CD3^+^ T cells **(A)**, CD4^+^ T cells **(B)**, CD8^+^ T cells **(C)**, and CD20^+^ B cells **(D)** were determined by FACS analysis and presented as % of total human leukocytes in PBMC. Statistical significance of comparison was determined using the paired t test.


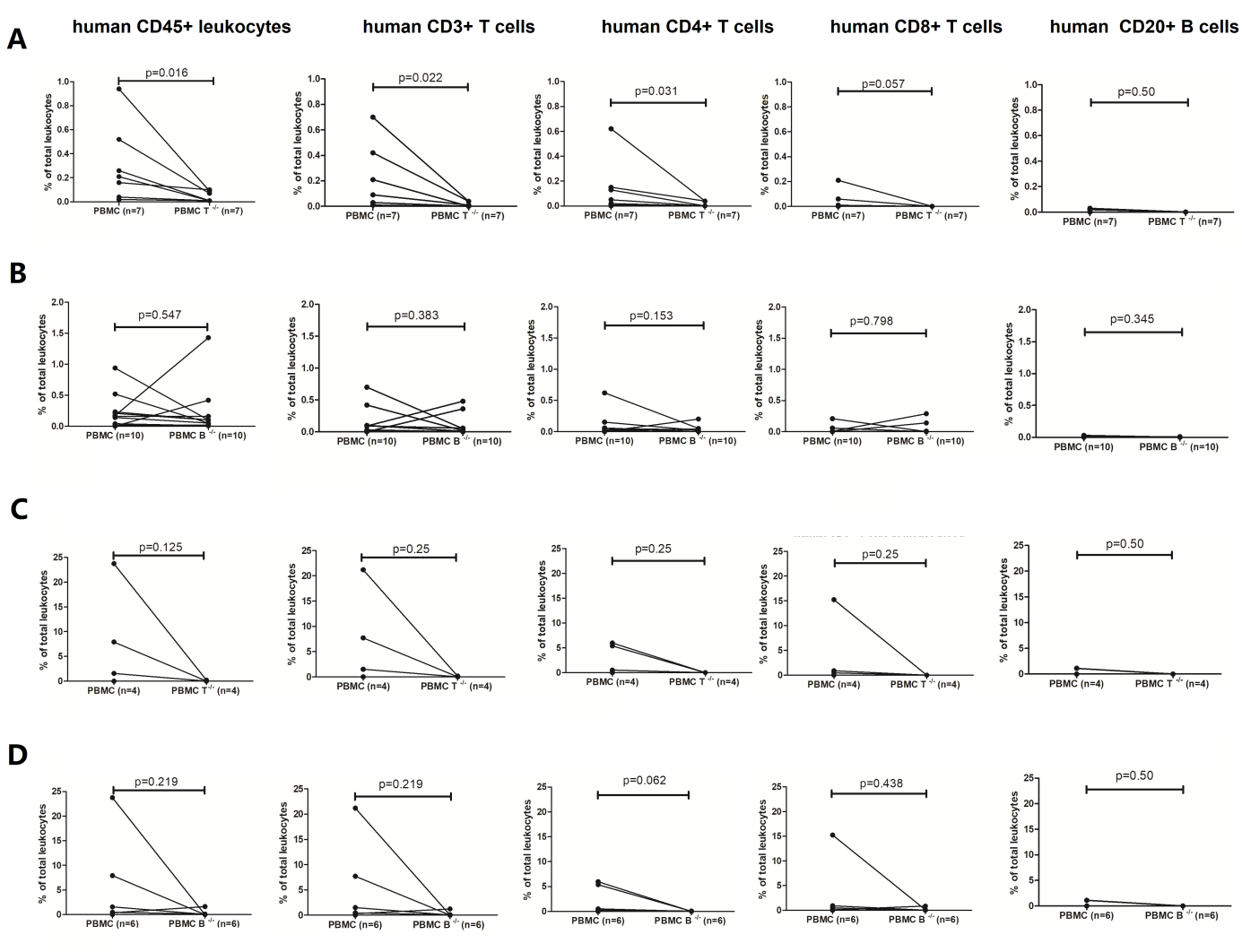


**Supplementary figure 2. Levels of human leukocytes in peripheral blood of recipient mice.** Mice were transferred with whole PBMC, T-cell depleted, or B-cell depleted PBMC, and peripheral blood was taken at the 6^th^ week (A,B) and 12^th^ week (C,D) after the transfer. Subsequently, human CD45^+^ leukocytes, CD3^+^ T cells, CD4^+^ T cells, CD8^+^ T cells and CD20^+^ B cells were identified by flow cytometry. Levels of human leukocytes are presented as percentage of total leukocytes including murine and human leukocytes in murine blood. Comparison on levels of human leukocytes indicated between mice transferred with whole PBMC (n=7) and mice transferred with T-cell depleted PBMC (n=7) (A) and between mice transferred with whole PBMC (n=10) and mice transferred with B-cell depleted PBMC (n=10) (B) by week by the 6^th^ week after the transfer. Comparison on levels of human leukocytes indicated between mice transferred with whole PBMC (n=4) and mice transferred with T-cell depleted PBMC (n=4) (C) and between mice transferred with whole PBMC (n=16) and mice transferred with B-cell depleted PBMC (n=6) (D) by week by the 12^th^ week after the transfer. P values reflect comparisons between mice transferred with whole PBMC and mice transferred with T- or B-cell depleted PBMC. Statistical significance was determined using the Wilcoxon matched pairs test.

**
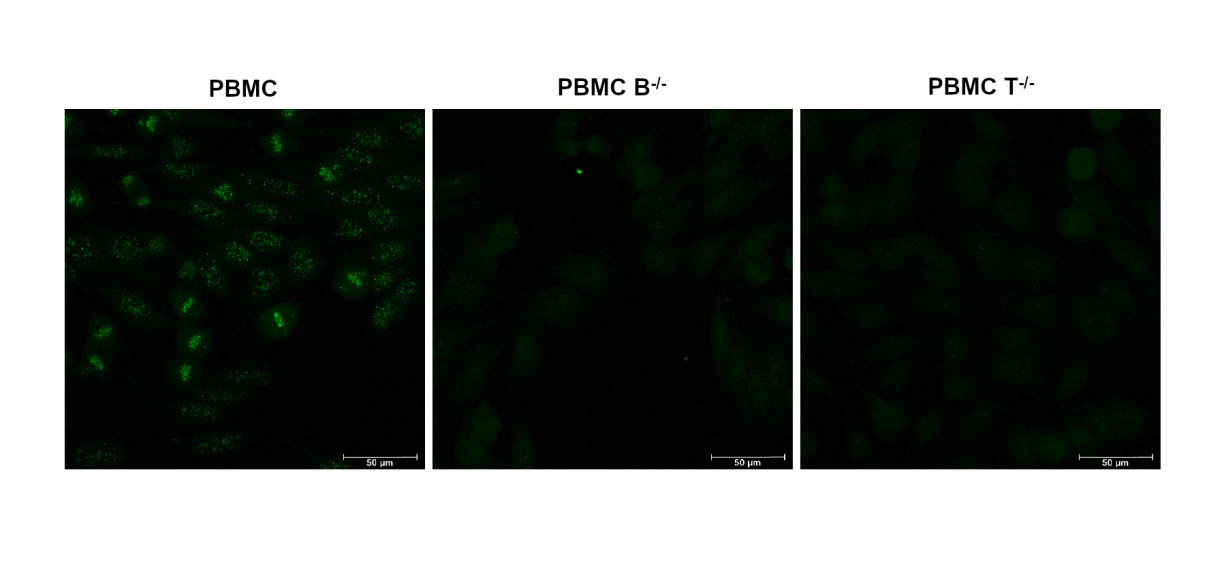
**

**Supplementary figure 3. Presence of ANA in sera of PBMC-transferred mice.** Antinuclear antibody (ANA) pattern of murine sera were detected using (HEp-2) cell-based immunofluorescence staining (EUROPattern Suite, Euroimmun, Germany). Two out of 7 mice which received whole PBMC from SSc patients scored positive for ANA, and their ANA patterns were consistent with those of two corresponding SSc patients. By contrast, mice which received T cell-depleted or B cell-depleted PBMC isolated from the 2 SSc patients were ANA negative. Representative micrographs of the ANA test for mice received whole PBMC, T cell-depleted PBMC (PBMC T^-/-^) or B cell-depleted PBMC (PBMC B^-/-^) are shown.

**
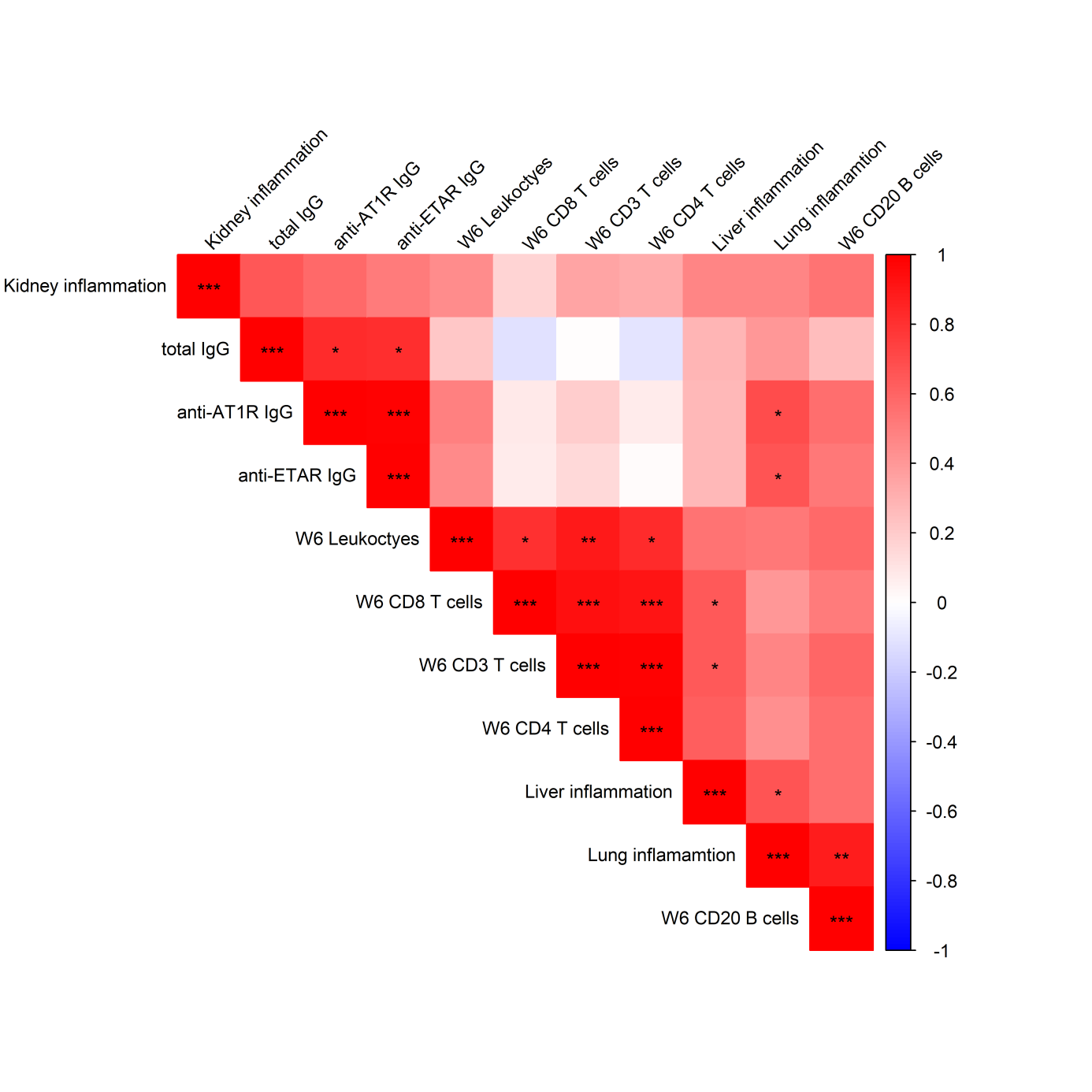
**

**Supplementary figure 4. Matrix of Spearman’s correlation coefficients between immunological and histopathological variables.** Severity of inflammation in the lung, kidney and liver as well as levels of circulating human CD45^+^ leukocytes, CD3^+^ T cells, CD4^+^ T cells, CD8^+^ T cells and CD20^+^ B cells at 6^th^ week after the cell transfer, and levels of total IgG, anti-AT1R IgG and anti-ETAR IgG in sera obtained after the sacrifice of mice were used for the analysis. Samples were tested for normal distributionby using Shapiro-Wilk normality test. Since most variables were not normally distributed, Spearman correlation was applied for the analysis. A color-coded correlation scale is provided on the right of the plot. Blue and ellipses represent negative and positive correlations, respectively, and darker color tones representd larger correlation coefficient magnitudes. Statistically significant differences are indicated by asterisks (**p*<0.05, ***p*<0.01 and ****p*<0.001).
